# Do cell culturing influence the radiosensitizing effect of gold nanoparticles: a Monte Carlo study

**DOI:** 10.1186/s40658-025-00746-3

**Published:** 2025-04-18

**Authors:** J. Antunes, T. Pinheiro, I. Marques, S. Pires, M. Filomena Botelho, J. M. Sampaio, A. Belchior

**Affiliations:** 1 Laboratório de Instrumentação e Física Experimental de Partículas, Av. Prof. Gama Pinto 2, Lisboa, 1649-003 Portugal; 2 Departamento de Física da Faculdade de Ciências da Universidade de Lisboa, Rua Ernesto de Vasconcelos, Lisboa, 1749-016 Portugal; 3 Departamento de Engenharia e Ciências Nucleares, Instituto Superior Técnico, Universidade de Lisboa, Estrada Nacional 10, Bobadela LRS, 2695-066 Portugal; 4 iBB– Instituto de Bioengenharia e Biociências, Instituto Superior Técnico, Universidade de Lisboa, Av. Rovisco Pais 1, Lisboa, 1049-001 Portugal; 5 Coimbra Institute for Clinical and Biomedical Research (iCBR) Area of Environment Genetics and Oncobiology (CIMAGO), Institute of Biophysics, Faculty of Medicine, Univ Coimbra, Coimbra, 3000-548 Portugal; 6 Center for Innovative Biomedicine and Biotechnology (CIBB), Univ Coimbra, Coimbra, 3000-548 Portugal; 7 Clinical Academic Centre of Coimbra (CACC), Coimbra, 3004-561 Portugal; 8 Centro de Ciências e Tecnologias Nucleares, Instituto Superior Técnico, Universidade de Lisboa, Estrada Nacional 10, Bobadela LRS, 2695-066 Portugal

**Keywords:** Gold nanoparticles, Radiosensitizers, Monte Carlo simulation, Realistic cell model, Cell culturing, Direction of irradiation, Cell survival fraction

## Abstract

**Background:**

Cell culture can be categorized into two major types: adherent and suspension. Both are used in a range of diverse research applications, exhibiting *Pros and Cons*, depending on what is being studied. In the field of Internal Emitters (IE), different morphological features such as nuclei size, cytoplasm ratio, and shape could influence its non-uniformity deposition and thus impact on the biological outcome. In this work we tested the hypothesis that cellular morphology differences, offered by adherent and suspension cultures, influence the radiosensitizing effect of gold nanoparticles (AuNPs).

**Methods:**

Using two PC3 cellular models, taken using confocal microscopy, we conducted Monte Carlo simulations to investigate the effects of different irradiation conditions on cellular Survival Fractions (SF). Our simulations focused on cells exposed to two distinct irradiation sources: ^60^Co and 14 MeV protons, along both the longer and shorter axes of the cells to assess directional influences on cell survival. Additionally, we compared the SF of cells adherent to the culture flask with those in suspension, reflecting different experimental and potentially clinical scenarios.

**Results:**

In the absence of AuNPs, neither cell type nor irradiation direction significantly affected SF for the radiation types tested. However, with AuNPs present, SF demonstrated a strong dependence on irradiation direction and cell morphology.

**Conclusions:**

Our results indicate that the direction of irradiation plays a crucial role in determining the effectiveness of AuNPs in reducing SF. Furthermore, the results suggest that using cells in suspension will reduce the dependence of cell survival on the beam direction during irradiation, regardless of the radiation quality used.

**Supplementary Information:**

The online version contains supplementary material available at 10.1186/s40658-025-00746-3.

## Background

Radiosensitization (RS) involves enhancing the effects of radiation through physical, chemical, or pharmacological methods [1]. Radiosensitizing agents function at the cellular level by employing different mechanisms, such as suppressing cell growth, preventing DNA damage repair, and generating toxic substances through radiolysis [2, 3].

Macromolecules of varying sizes, small molecules, or specific types of nanoparticles (NPs) can work as radiosensitizers agents. Several studies have provided both experimental and theoretical evidence for the RS effects of metallic nanoparticles [4, 5]. The presence of high-Z nanoparticles, such as gold nanoparticles (AuNPs), near tumor cells can enhance photon absorption and increase the production of secondary electrons, including photoelectrons, Auger electrons, and Compton electrons. The significant production of Auger electrons and low-energy delta-rays (typically in the range of tens of keV) plays a crucial role in enhancing radiosensitivity in the surrounding microenvironment [6]. Furthermore, ionization electrons generated when secondary electrons (delta rays) undergo further interactions within high-Z nanoparticles contribute to this effect. When combined with protons, the secondary electrons are primarily ejected from the outer atomic layers, resulting in a small proportion of Auger electrons [5]. However, experiments combining charged particles with AuNPs have shown significant effects, underscoring the need for further investigation into the radiosensitizer effect of AuNPs when used with this type of external radiation [7].

Recent studies have demonstrated the potential of radiosensitizers to increase the efficiency of radiotherapy in various types of cancer, including glioblastoma, lung cancer, breast cancer and prostate cancer [8–11].

Additionally, studies have shown that cell morphology and adhesion can affect radiosensitivity [12]. They evaluate how the radiosensitivity of the cells was influenced by cell culturing under various treatments, including boron neutron capture therapy, neutrons, and gamma rays (γ-rays), concluding that adherent cells exhibit higher radiosensitivity compared to suspended cells. This difference in sensitivity highlights the importance of considering cell culture conditions when interpreting results. When working with radiosensitizers, as they increase the production of secondary particles with short range, it is important to evaluate how different conditions, regarding the cell and the irradiation setup, can lead to different cell radiosensitivity. A recent study, using 3D pancreatic cancer co-culture models, demonstrated a remarkable radiosensitization effect with AuNPs, highlighting the potential impact of cell organization on treatment response [13].

Therefore, understanding how cell culturing protocols affect AuNP-mediated radiosensitization is crucial for optimizing their application in cancer therapy. Recent studies investigated how manipulating factors, like AuNP size and surface functionalization, can be used to achieve optimal radiosensitization [14]. However, there is still a need for a more detailed investigation into how cell culturing conditions influence this effect. Specifically, it is important to understand whether the state of the cells (adherent vs. suspension) and the irradiation method (type and geometry) affect the radiosensitizing efficiency of AuNPs.

To address these gaps, we conducted Monte Carlo simulations to investigate the effects of different irradiation conditions on cell radiosensitivity. Our simulations focused on cells exposed to two distinct irradiation sources: ^60^Co, a widely used gamma-ray source in clinical radiotherapy, and 14 MeV protons, since these are two sources that we have available in the group and are frequently used in cellular irradiation. We simulated irradiation applied along both the longer and shorter axes of the cells to assess directional influences on cell survival. Additionally, we compared the survival fractions of cells adherent to the culture flask with those in suspension, reflecting different experimental and potentially clinical scenarios. By integrating the use of AuNPs as radiosensitizers into our simulations, we aimed to determine how these agents modulate radiosensitivity under the various conditions described.

## Methods

### Acquisition of confocal microscopy images, of PC3 cells, used in the MC simulations

PC3 cells were seeded at a density of 80,000 cells per coverslip in 500 µL of medium and left in the incubator at 37ºC in a humidified atmosphere of 5% CO_2_.for 24 h to allow for adherence. Then, cells were stained with 1 µL of CytoPainter Green Fluorescence (Abcam, ab176735) and incubated for 30 min at 37 °C, protected from light. Afterwards, live cell images were acquired in a LSM 710 Axio Observer Z1 confocal laser scanning microscope, equipped with QUASAR detection unit and ZEN Black software (all from Carl Zeiss, Germany), using a Plan-Apochromat 20X/0.8 M27 and Argon/2 (488 nm) laser. Z-stacks were acquired by using an interval of 17.45 μm.

### MC studies

MC simulations were performed using TOPAS (TOol for Particle Simulation; version 3.8) [15], which is layered on top of Geant4 [16], version v10.07.p03. To study the influence of cell culturing, two realistic cell models—adherent and suspension—were constructed from confocal microscopy images (see Sect. 2.1) and implemented in the MC code. The implementation of the 3D realistic cell models was initiated by acquiring a z-stack of confocal microscopy images, as previously outlined. Then the confocal microscopy z-stack underwent preprocessing to enhance quality. Subsequently, ImageJ software [17] was employed for segmentation, facilitating the distinction between the cytoplasm and the nucleus in the images. To streamline the association of specific tag numbers with each component, a Python script was developed. This script read the segmented images from ImageJ, assigning tag numbers to voxels, and generated a binary file utilized as input in TOPAS. The detailed methodology is described elsewhere [14].

#### Validation of the cellular SF MC model

**Fig. 1:**
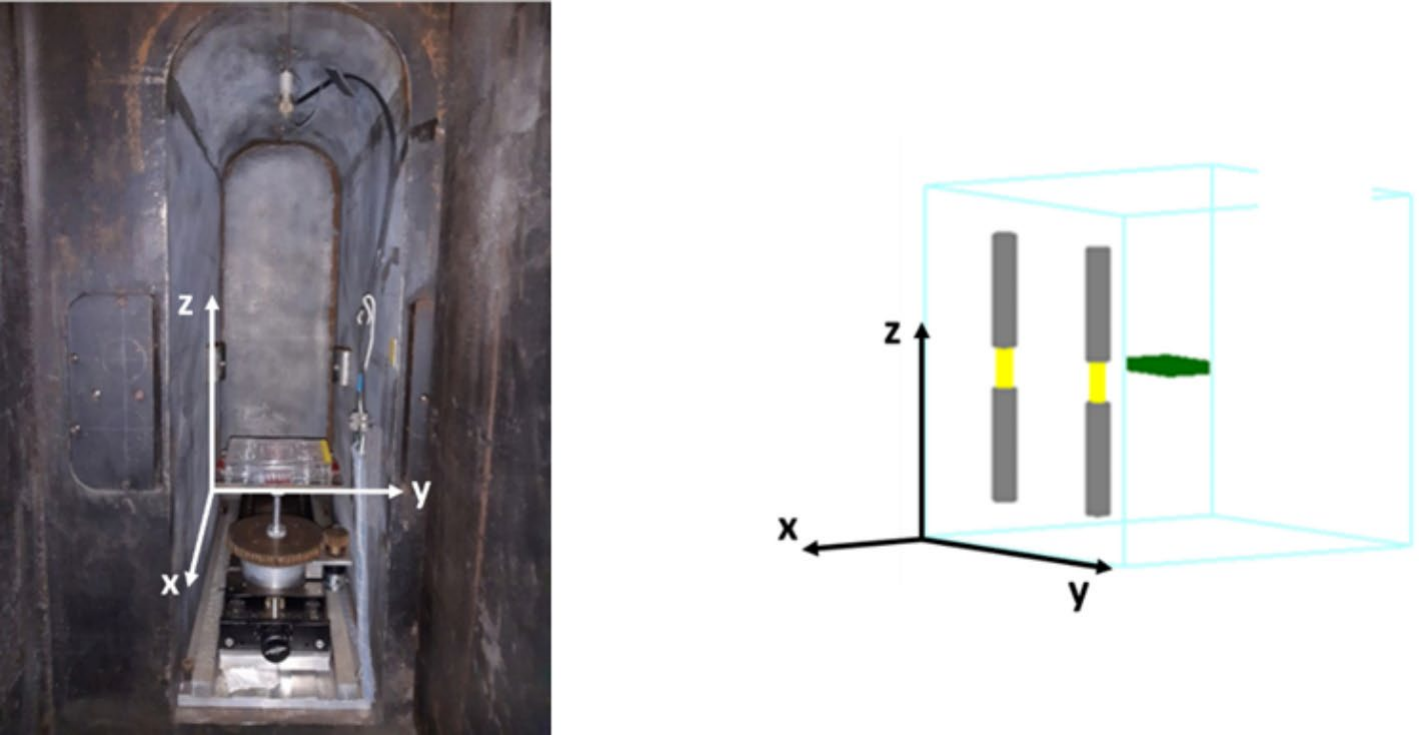
**Left**: The inside of Precisa-22. **Right**: Irradiation setup implemented on TOPAS: Two sources: grey bars represent the stainless-steel cylinders, yellow cylinders represent the ^60^Co sources, and green represents the 96-well plate

The radiosensitizing effect of AuNPs under various cell culture conditions and beam irradiation setups was assessed using SF calculations, described in the sequence (Sect. 2.2.4). So, the first step includes the validation of the cellular SF MC model, against experimental data (see Supplementary Information). For that, the irradiation setup was designed to replicate the PRECISA-22 ^60^Co irradiator (Fig. 1.) used in radiobiological experiments. This irradiator has four sources enclosed in stainless steel cylinders, but only two have significant activity. The walls of the irradiator were made of lead. While the lead walls can contribute to the production of secondary scattered radiation, they were not explicitly included in the MC simulation. The exclusion of the walls from the simulation may lead to an underestimation of scattered radiation contributions, but this choice was made to simplify the computational model while focusing on the primary interactions within the AuNPs. Therefore, our simulation included only the two active ^60^Co sources and accurately modeled the dimensions of the irradiator chamber and the position of the 96-well plate inside it when simulating its irradiation. The source was chosen to be a discreate spectrum of photons with energies of 1.17 and 1.33 MeV with equal weights. From the simulation, a phase space file was recorded on the surface of the 96-well plate and was then used as a source to irradiate each cellular computational model.

#### Study on the impact of ^60^Co beam irradiation setup on cellular SF

In addition to the effects of different cell culturing on cellular SF, the beam direction may also significantly influence the production of short-ranged electrons around AuNPs, thereby impacting cellular SF. Therefore, two additional sets of simulations were conducted.

In the first one, the simulation setup consisted in a ^60^Co beam, along the z-axis, perpendicular to the cell plane. The distance between the source and the cell surface was established at 0.5 μm, with a homogeneous incident photon field. The γ-rays were emitted from a flat, rectangular plane source, with dimensions matching those of the reconstructed cell model. This setup was chosen to ensure uniform irradiation across the entire model, simulating realistic exposure conditions. In the second set of simulations, all parameters remained the same except for the direction of irradiation, which was along the x-axis.

#### Study on the impact of 14 mev proton beam irradiation setup on cellular SF

Driven by the increasing interest in the use of radiosensitizers in Proton Therapy, additionally to ^60^Co gamma-radiation, we modelled a 14 MeV proton beam at the Bragg peak (at a depth of 0.3 cm in water) along the z-axis and x-axis. To achieve this, a simulation was first performed to irradiate a water volume with a 0.5 cm side using 14 MeV protons, and a phase space file was recorded at the Bragg peak. This phase space file was then transversely resized to match the dimensions of each PC3 cellular computational model and used as the source for their irradiation.

#### MC code parameters and AuNPs specifications

For all the irradiations described, to simulate the physical interaction between radiation and matter, the Geant4-DNA physics list was used. However, this list is only compatible with water as a material. Therefore, the Livermore physics list was used to incorporate the AuNPs, as it is the physics list that offers the best performance at nanoscale electron transport [18].

The secondary electron production limit was defined as 10 eV [19, 20]. The production of fluorescence and Auger electrons and the Auger Cascade in the simulations were activated [21]. The range cut for electrons and gammas was set to 1 nm and the tracking step size to half the AuNP diameter (4.79 nm).

To ensure a balance between computational efficiency and uncertainties, a consistent quantity of 1 × 10^7^ primary particles was employed. Ten separate simulations were executed under the simulated conditions, each assigned a distinct seed number, resulting in a cumulative total of 1 × 10^8^ primary particles. This particle count yielded a dose uncertainty below 5%.

Several conditions were simulated. First, the presence of AuNPs was not considered. Then, AuNPs with a diameter of 4.79 nm were placed in the cell with different distributions. Their percentage within the nucleus was changed from 0% (when all AuNPs were uniformly placed only in the cytoplasm) to 100% (when all the AuNPs were uniformly placed inside the nucleus). The total number of AuNPs in the cell was estimated to be 4326, considering the cell uptake and the mass of one AuNP ($\:m=1.11\times\:{10}^{-9}\:$ ng). The size and the uptake of AuNPs were determined in a previous study(Marques et al., 2022). The position of each AuNP was determined as reported in our previous work [14]. Briefly, a Python script was used, with input parameters including the number and size of the NPs, voxel dimensions, and the cellular model dimensions. Random voxels, each matching the size of an AuNP, were selected, ensuring that the chosen voxel belonged either to the cytoplasm or the nucleus, depending on the scenario being simulated. If all AuNPs were placed inside the nucleus, only nucleus voxels were selected, and similarly for the cytoplasm.

### Calculation of SF

The deposited energy in each voxel outside the AuNP was obtained by simulations. The survival fraction, $\:SF,$ was obtained by

$$\:SF={e}^{-\stackrel{-}{L}},$$

where $\:L$ is the mean number of lesions in the volume of the cell nucleus, obtained in the framework of the Linear Quadratic Model (LQM). The average number of lesions with and without the presence of AuNPs was calculated using

$$\:\stackrel{-}{L}=\alpha\:D+\beta\:{D}^{2},$$

where $\:D$ is the mean dose in the nucleus, and $\:\alpha\:$ and $\:\beta\:$ are the parameters of the LQM.

$$D = {1 \over M}\sum\limits_{i = 1}^n {{ \in _i}} $$

with $\:n$ representing the number of voxels belonging to the nucleus volume and $\in _i$ the deposited energy in each of those voxels and $\:M$ is the total mass of the nucleus.

In the presence of AuNP, we computed additionally the $\:SF$ using a version of the Local Effect Model (LEM), where

$$\:\stackrel{-}{L}=\frac{1}{n}\sum\:_{i=1}^{n}{L}_{i}.$$

The $\:{L}_{i}$ represents the number of lesions in each voxel and is calculated also in the framework of the LQM,

$$\:{L}_{i}=\left\{\begin{array}{c}\alpha\:{d}_{i}+\beta\:{d}_{i}^{2},\:{d}_{i}\le\:{D}_{t}\\\:\left(\alpha\:+2\beta\:{D}_{t}\right){d}_{i}-\beta\:{D}_{t}^{2},\:{d}_{i}>{D}_{t}\end{array}.\right.$$

{D}_{t}\end{array}.\right.]]>

Here $\:{d}_{i}$ corresponds to the deposited dose in each voxel and $\:{D}_{t}$ is the threshold dose, above which the LQM fails, and the dose-dependence becomes linear. That is, the LEM assumes that the number of lesions only depends on the dose deposited locally, in each voxel, and not on the type of the radiation. The effect the type of radiation on the $\:SF$ arises from the spatial distribution of the deposited energy inside the volume of the nucleus. The *α* and β parameters were obtained by fitting the experimental data for the reference radiation to the LQM.

Since our MC simulation results were calculated per primary particle, the mean dose in the nucleus ($\:D$) and the dose in each voxel ($\:{d}_{i}$) were multiplied by the experimental number of incident particles $\:N$ in a single cell. For the ^60^Co irradiation, this value was calculated considering the activity $\:\left(A\right)$ of the ^60^Co source used to perform the irradiation on the day of the experiment and the irradiation time $\:\left(t\right)$. It was scaled down to the cell dimensions as follows:

$$\:N\approx\:\left(\frac{{S}_{cell}}{{S}_{plate}}\right)\cdot\:t\cdot\:A.$$

Here $\:{S}_{plate}=57.63\:\text{c}{\text{m}}^{2}\:$is the cross-sectional area of the cell culture plate used in the experiments and $\:{S}_{cell}$ corresponds to the cross-sectional area of the simulated cell. The values for each source direction and cell model are presented in Table 1. For the proton irradiation, the number of incident particles was calculated considering the proton flux $\:(7.25\times\:{10}^{6}\:\text{p}\text{r}\text{o}\text{t}\text{o}\text{n}\:{\text{s}}^{-1}\text{c}{\text{m}}^{-2})$ instead of the ^60^Co activity.

**Table 1:** – Cross-sectional area $\:\left(\text{c}{\text{m}}^{2}\right)$ of the simulated cell models for the three simulated source directions

Source direction Cell model	z-axis	x-axis	2 sources
Adherent	$\:3.30\cdot\:{10}^{-6}$	$\:1.67\cdot\:{10}^{-6}$	$\:1.10\cdot\:{10}^{-5}$
Suspension	$\:2.35\cdot\:{10}^{-6}$	$\:2.46\cdot\:{10}^{-6}$	$\:5.07\cdot\:{10}^{-6}$

## Results

### MC geometry - adherent and suspension cells phantoms

**Fig. 2:**
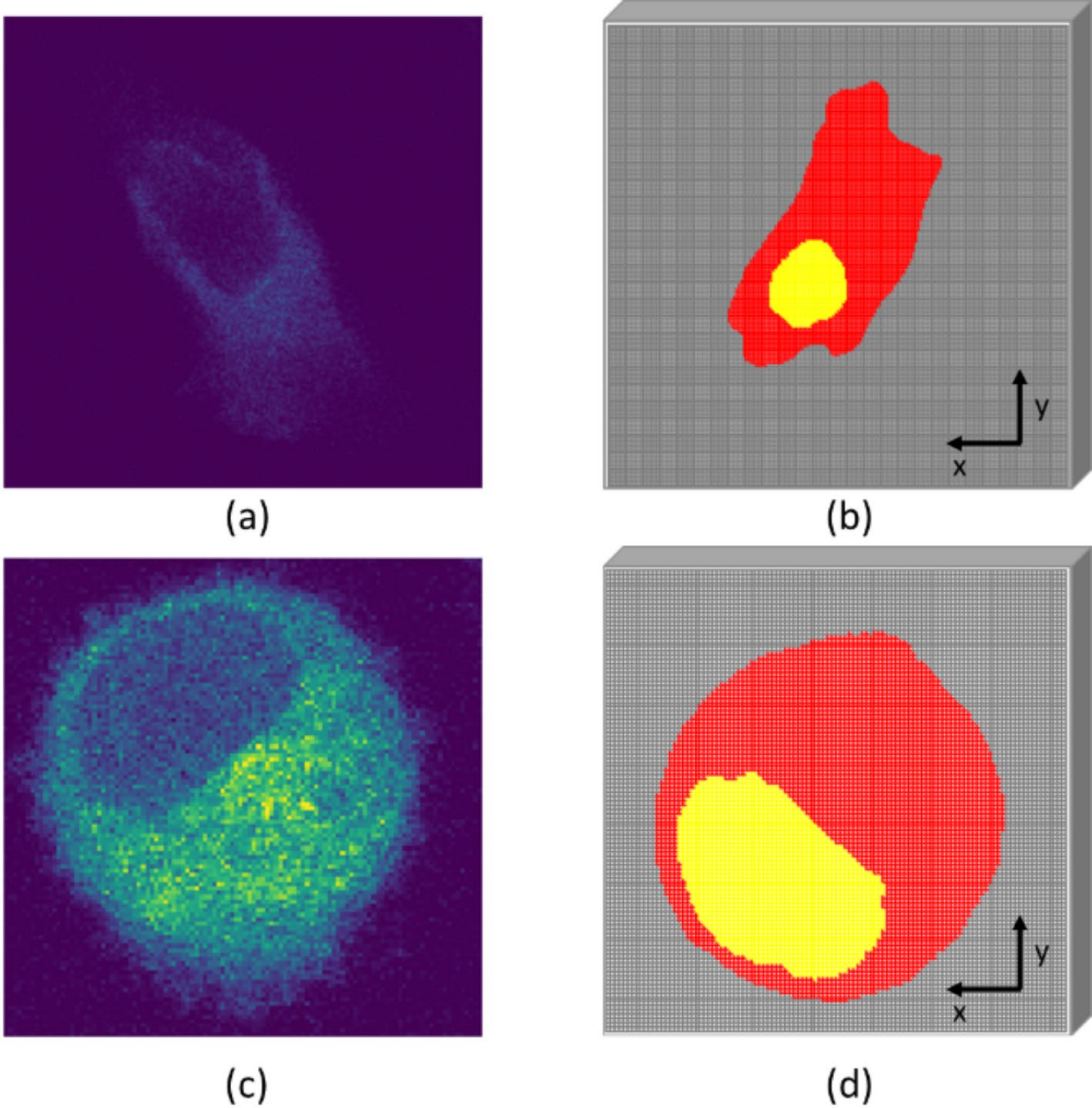
Realistic cell geometry modeling. Top images show **(a)** the 2D confocal microscopy image and **(b)** the reconstructed cell phantom defined in TOPAS for the adherent cell culture (voxel dimensions of 0.21 × 0.21 × 0.47 $\:\mu\:{m}^{3}$). Bottom images show **(c)** the 2D confocal microscopy image and **(d)** the reconstructed cell phantom defined in TOPAS for the suspension cell culture (voxel dimensions of 0.21 × 0.21 × 0.60 $\:\mu\:{\text{m}}^{3}$). In both reconstructed cell phantoms in yellow are illustrated the nucleus and in red the cytoplasm. The images are not at scale

The central slice of the voxelized cell phantoms implemented in TOPAS, and the respective original confocal microscopy image are shown in Fig. 2, for both cell culture types. The top images illustrate the result obtained when the cell is adherent to the culture flask and the bottom images show the results when the cell is in suspension. Despite the reconstructed cell models appearing rotated relative to the original confocal images, this is merely a visualization issue and does not impact the simulation results. From the morphological point of view, the cell in suspension exhibits a more spherical form compared with the adherent cell, where the form is more irregular and flatter. The nucleus volumes are 281 μm³ and 420 μm³, while the cytoplasm volumes are 2502 μm³ and 3587 μm³ for adherent and suspension cells, respectively.

### Calculation of the SF using the MC model for both adherent and in suspension cellular phantoms, post-^60^ Co irradiation

#### Without AuNPs

**Fig. 3:**
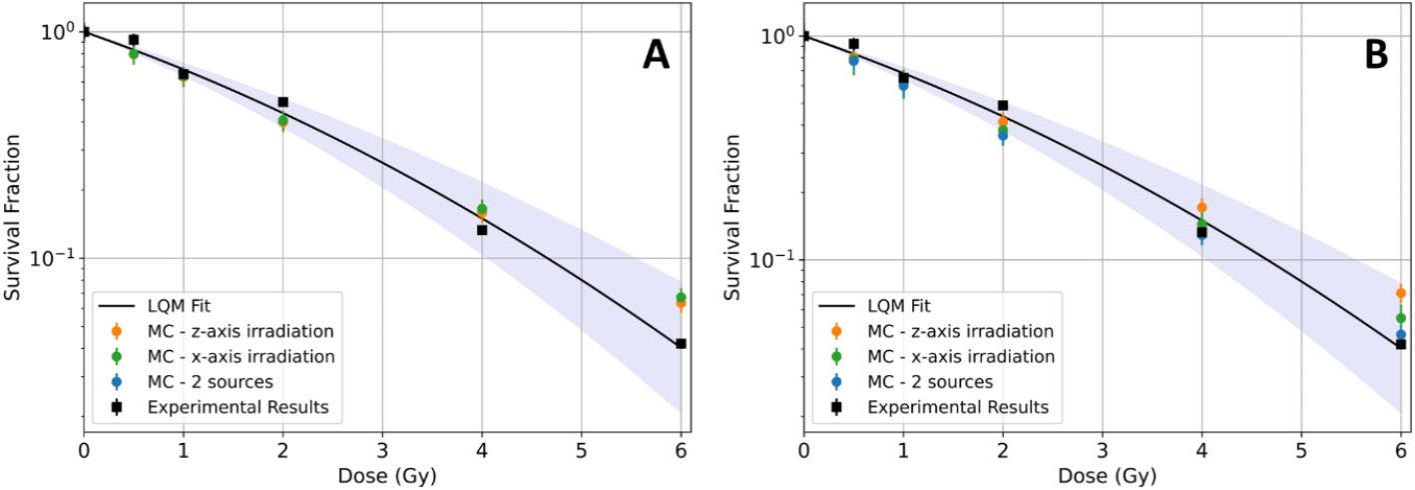
Comparison of the experimental survival fraction (black) with the one obtained by MC simulation, in the absence of AuNPs, for the (**A**) suspension and (**B**) adherent cell culture for all the simulated irradiations, 2 sources (blue), along the z-axis (orange) and x-axis (green). The region of statistical uncertainty of the LQM fit is illustrated (shaded area) as well

The experimental SF curve in the absence of AuNPs (see Supplementary Information) was compared with the SF curves obtained using MC simulations for both cell geometries, in suspension (see Fig. 3A) and adherent (see Fig. 3B) and for the different irradiation setups (2 sources and along z-axis and x-axis). The 2 sources irradiation replicates the experimental conditions (Fig. 1B). In the z-axis irradiation, each cell model is irradiated perpendicular to its plane, along the z-axis, which means the radiation is directed vertically through the cell. In the x-axis irradiation, the cells are irradiated along the x-axis, which means the radiation is applied parallel to the cell plane.

The primary finding of this study, excluding the use of AuNPs, is that no significant differences were observed between experimental results and the SF curve obtained using the modeling of 2 sources for both adherent and suspension cells. Our findings align with those of Cansolino et al. [12], who also observed no significant differences when cells were irradiated with doses up to 6 Gy, with ^60^Co. Therefore, we use the experimental SF curve for PC3 adherent cells to validate both cellular MC models.

When comparing the two cellular models irradiated along the z-axis (shown in orange), the results indicate a decrease in SF for the cells in suspension compared to the adherent ones, with differences ranging from 2 to 15% for the lower and higher doses simulated. This can be due to the difference in the size of the cell nucleus in both models, being the nucleus of the cell in suspension bigger and less flat than the nucleus of the adherent cell. Consequently, when irradiated along the z-axis, the larger nucleus of the suspension cell model makes it more susceptible to damage, leading to a lower survival fraction compared to the adherent cell model. The contrary happens when the irradiation was performed along the x-axis, where the SF calculated using the suspension cell model is higher than the one obtained when the adherent cell model is used. Here, the adherent cell model’s flatter shape makes it more vulnerable to damage, resulting in a lower SF compared to the suspension cell model.

These findings are highly significant for bio-effect modeling, as they demonstrate the importance of considering cellular morphologies, particularly nuclear size, when attempting to standardize radiation-induced biological effects based on in vitro experiments.

#### With AuNPs

The following studies were pursued using a cellular dose of 2 Gy for three main reasons: (i) it is the dose per fraction in radiation therapy; (ii) at 2 Gy, Fig. 3 shows no significant differences among the different scenarios (cell morphology and beam directions), allowing us to eliminate certain uncertainties by using the data without AuNPs as a control; and (iii) the experimental SF curve performed in our previous work [22] was for 2 Gy ^60^Co radiation.

**Fig. 4:**
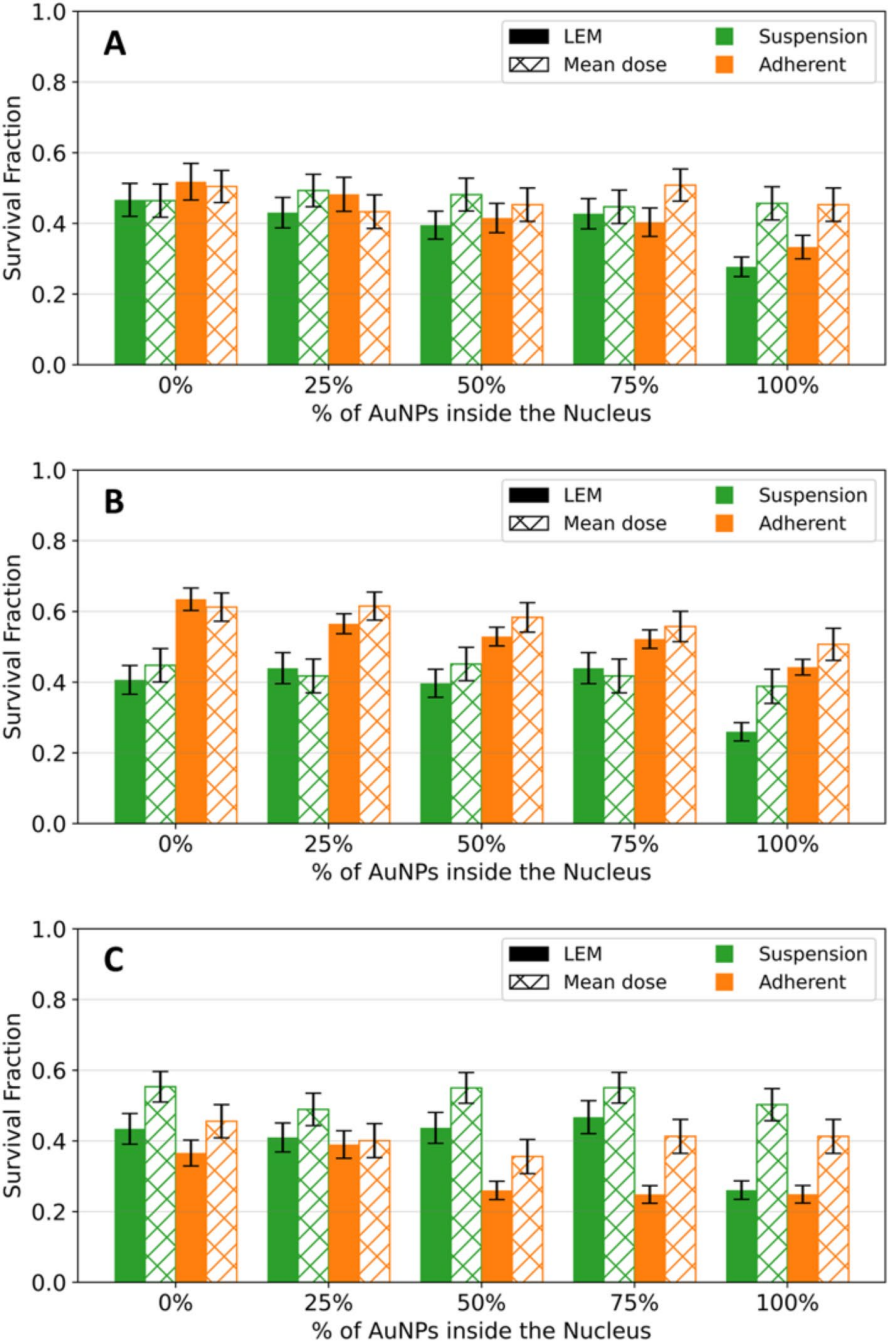
- Comparison of the SF obtained by MC simulation for a 2 Gy ^60^Co radiation, in the presence of AuNPs, for the suspension (green) and adherent (orange) cell cultures. (**A**) Represents the results with 2 sources, (**B**) along the z-axis and (**C**) along the x-axis of irradiation. The solid bars represent the *SF* obtained using the LEM and the patterned bars illustrate the *SF* obtained by the mean dose approach

The SF MC model was performed in the presence of 4326 AuNPs distributed inside both cell phantoms, for 2 Gy of ^60^Co irradiation. The survival fractions were simulated for different percentages of AuNPs inside the nucleus (0, 25, 50, 75 and 100%) and the results, for both mean dose and LEM approaches, are shown in Fig. 4.

The LEM approach predicts more accurate survival fractions by directly accounting for lethal lesion formation at a voxel level, which includes the effects of localized energy deposition around AuNPs. In contrast, the mean dose approach averages the total deposited energy over the entire nucleus, assuming a uniform dose distribution. This averaging mask the magnitude of localized events such as short-range electrons and the effect on local dose and local damage in the vicinity of AuNPs.

For all the irradiation setups and cell geometries, it is possible to conclude that the mean dose approach underestimates cell damage due to AuNP-enhanced localized effects.

Given this, the evaluation of how cell culturing impacts the radiosensitizing effect of AuNPs was conducted using survival fractions obtained with the LEM approach.

In line with the results obtained without AuNPs, no significant differences are obtained for the SF obtained in both cells morphologies (adherent and in suspension) when the setup of irradiation includes the use of two ^60^Co sources (see Fig. 4A). From our previous work [22], there was a significant loss in cellular proliferation ability for PC3 cells exposed to 2 Gy of γ-radiation after incubation with AuNP-BBN. The observed survival fraction was 46.6 ± 1.2% and 2.7 ± 1.78 for irradiation only or for irradiation combined with AuNP-BBN (gold nanoparticle functionalized with a thiolated bombesin peptide), respectively. Indeed, the MC model results in higher SF when compared to the experimental one.

**Fig. 5:**
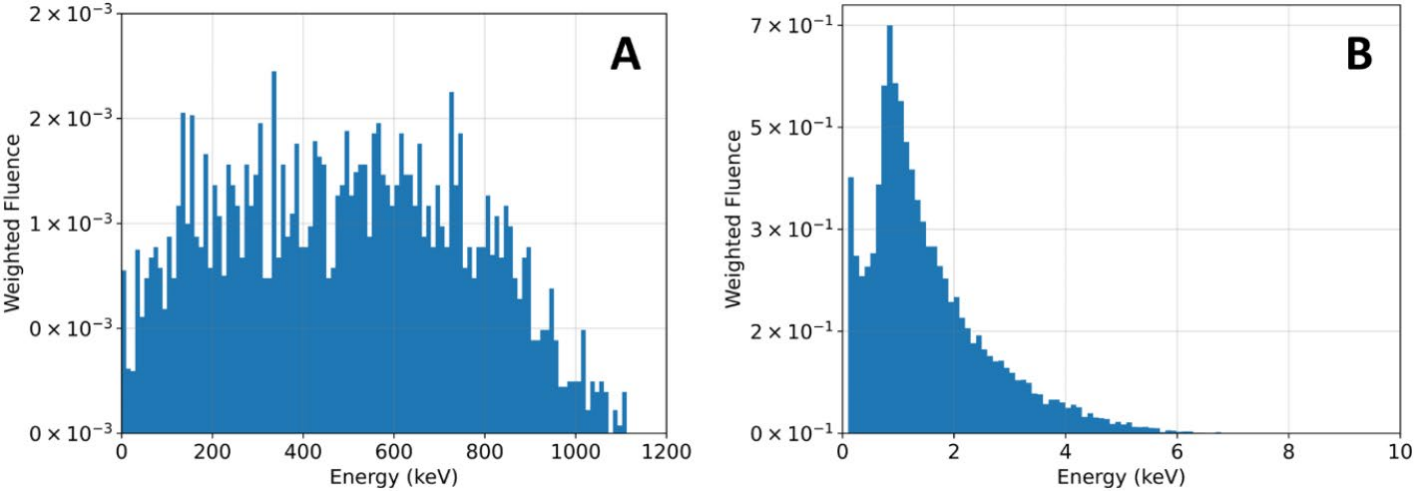
–Energy spectra of the secondary electrons produced from the interaction of **(A)**^60^Co γ-rays and **(B)** 14 MeV protons with the AuNPs

Without AuNPs, we observed that nuclei size affects the MC SF curves in the absence of AuNPs. To explore whether the same behavior occurs in the presence of AuNPs, we measured the SF values for irradiation along both the z-axis (Fig. 4B) and the x-axis (Fig. 4C). Along the z-axis (Fig. 4B), i.e. perpendicular to the cell, the SF is significantly higher in adherent cells, when compared with cells in suspension, for all % AuNPs inside the cell. Due to its characteristic interaction with the AuNPs, this effect cannot be solely attributed to the ^60^Co radiation source. The presence of AuNPs leads to additional energy deposition through the production of secondary electrons. Therefore, to deeply understand our results, we obtained the energy spectra (see Fig. 5A) of the electrons produced in the vicinity of the AuNPs.

The secondary electrons produced had an average energy of 490 keV, equivalent to a range in water of about 1 mm, with only a small percentage having a range in water of micrometers. In the low-energy electron component of the spectrum, the higher the energy the greater the number of electrons produced. Given that the adherent cell model has a flatter shape, its nucleus tends to be more elongated and thinner. Consequently, when cells are irradiated along the shorter axis (x-axis), there is a greater likelihood of electrons depositing their energy inside the nucleus compared to irradiation along the z-axis. This is because, due to the elongated shape of the nucleus, a higher part of low-energy electrons with micrometer-range will deposit their energy within the nucleus. However, this does not occur in the case of irradiation along the longer axis (z-axis), as the nucleus is thinner, reducing the probability of secondary electrons depositing their energy within the nucleus, since the number of electrons capable of doing so is reduced due to their short range (orthogonal views of the cell models are provided in the Supplementary Information to help visualize the 3D structure).

Based on the results obtained, it would be advantageous to evaluate an enhancement factor of survival at 2 Gy, instead of directly comparing the survival fraction of each irradiation. The enhancement factor of survival at 2 Gy was calculated as the ratio between the SF obtained in the absence $\:\left(S{F}_{w/o\:AuNPs}\right)\:$and in the presence of AuNPs$\:\:\left(S{F}_{w/\:AuNPs}\right)$:

$$\:Enhancement\:factor=\frac{S{F}_{w/o\:AuNPs}}{S{F}_{w/\:AuNP}}.$$

**Fig. 6:**
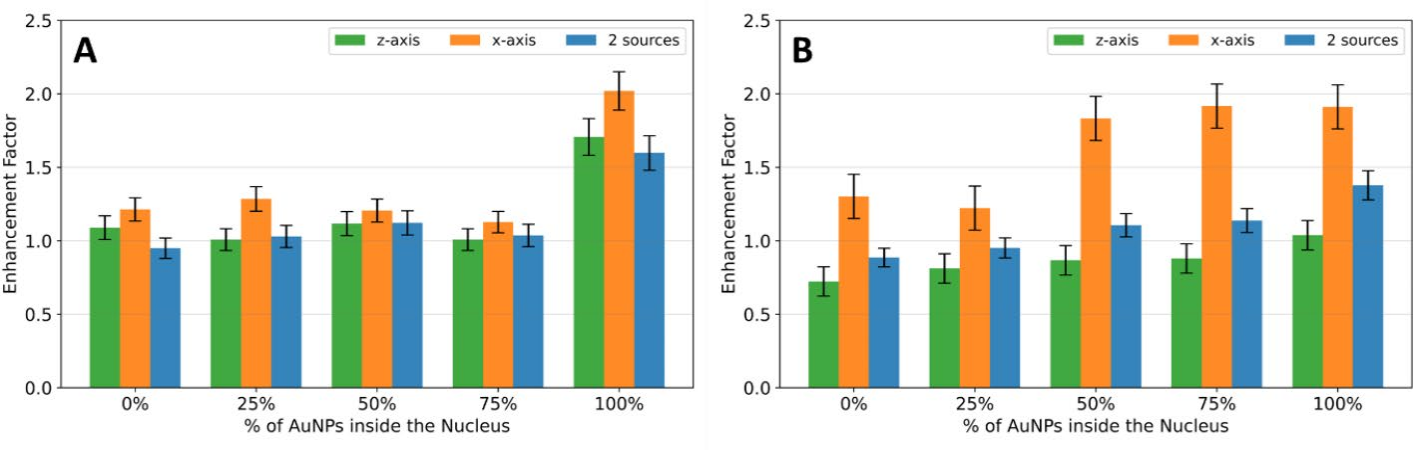
- Comparison of the survival fraction enhancement factor for a 2 Gy ^60^Co source irradiation, for the 2 sources (blue), along the z-axis (green) and x-axis (orange) irradiation, for the **(A)** suspension and **(B)** adherent cell culture

The results for both cell models are shown in Fig. 6.

In the case of the suspension cell model (Fig. 6A), when comparing the enhancement factor of the survival fraction at 2 Gy for each irradiation, higher values obtained when all the AuNPs are placed inside the nucleus are observed, but variations between different irradiation setups seem irrelevant. This outcome was expected since cells in suspension have spherical form and the area exposed to the beam is similar regardless of the direction of the beam. When comparing the results for all irradiations in the case of the adherent cell model (Fig. 6B), a significant variation is observed in the enhancement factor across all concentrations of AuNPs simulated within the nucleus. This effect is more significant when the cell model was irradiated along the x-axis. This difference is mainly justified by the variation of the exposed area of the cell to the beam and the beam direction.

For the energy spectrum presented in Fig. 5A, the secondary electrons have ranges up to 2 mm in water, which is much larger than cellular dimensions. Therefore, a more rigorous simulation should consider the interactions of the primary beam with nearby cells, culture medium, the container well, air and other surroundings that can affect the radiation field inside the cell. As such, the results presented here should only be analyzed qualitatively. However, it is important to emphasize that this issue is expected to affect also the experimental results when comparing adherent and suspension cell cultures. The former is fixed to the bottom of the well, made of a plastic material with a layer of cell culture medium on top, while in suspension the cells are detached inside vials.

### Calculation of the SF using the MC model for both adherent and in suspension cellular phantoms, post-14 MeV protons irradiation

For the 14 MeV protons, the charge particle equilibrium conditions are satisfied, since the secondary electrons produced (Fig. 5B) have a maximum energy of about 10 keV, which corresponds to a maximum range of approximately 5 μm, which is inferior to the cell dimensions.

**Fig. 7:**
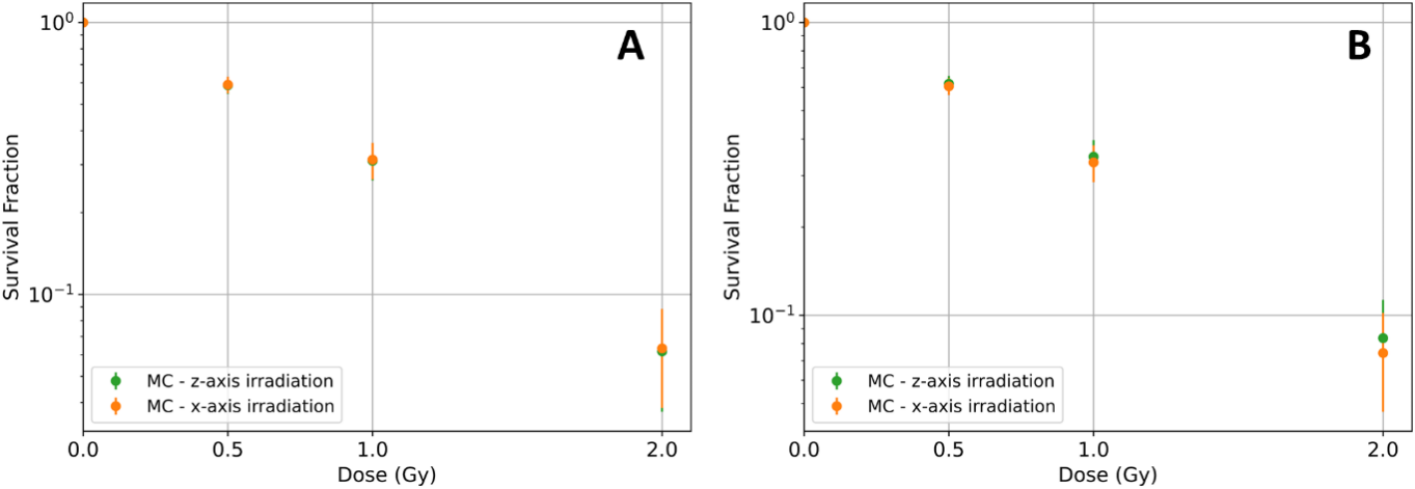
Comparison of the results obtained by MC simulation, in the absence of AuNPs, for the (**A**) suspension and (**B**) adherent cell culture for proton irradiation along the z-axis (orange) and x-axis (green)

The survival curves obtained, in the absence of AuNPs, using MC simulations for both cell geometries and proton irradiation setups were compared, in Fig. 7.

**Fig. 8:**
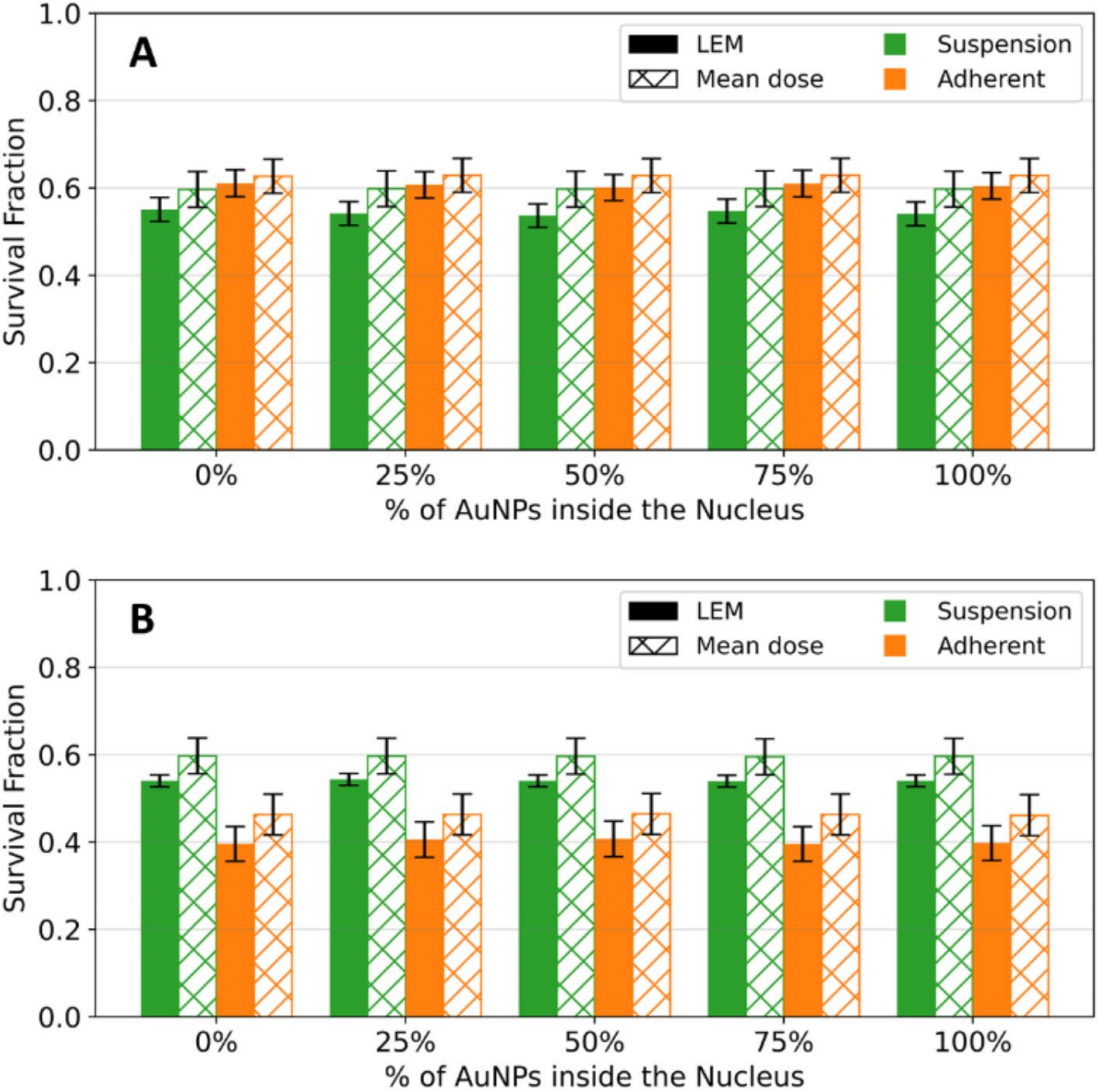
- Comparison of the survival fraction obtained by MC simulation for a 0.5 Gy proton along the **(A)** z-axis and **(B)** x-axis irradiation, in the presence of AuNPs (4326 NPs), for the suspension (green) and adherent (orange) cell culture. The solid bars represent the SF obtained using the LEM and the patterned bars illustrate the SF obtained by the mean dose approach

In the absence of AuNPs, the results obtained through MC simulations for the adherent and suspension cell models are similar regardless of the beam direction. When the presence of AuNPs was considered, the SF calculated using both approaches, mean dose and LEM, at 0.5 Gy for all the concentrations of AuNPs inside the nucleus and for both irradiations and models are shown in Fig. 8. This value of dose was chosen based on the survival fraction obtained in the absence of AuNPs.

In both irradiations, the SF at 0.5 Gy remain fairly constant across the different percentages of AuNPs, indicating that the survival fraction does not change significantly with the percentage of AuNPs inside the nucleus, regardless the cell model simulated. The SF calculated using both approaches are similar, which can be justified by the lower interaction probability between the AuNPs and the primary radiation, resulting in a small increase in the number of lethal lesions at a voxel level. Therefore, the presence of AuNPs does not significantly increase the production of lethal events locally, as the energy deposition by protons in the Bragg peak region is already near maximum, limiting additional dose enhancement effects from AuNPs.

For the suspension cell model, the results show that the cell survival obtained for the z-axis irradiation at 0.5 Gy are practically the same as the ones obtained for the irradiation along the x-axis.

**Fig. 9:**
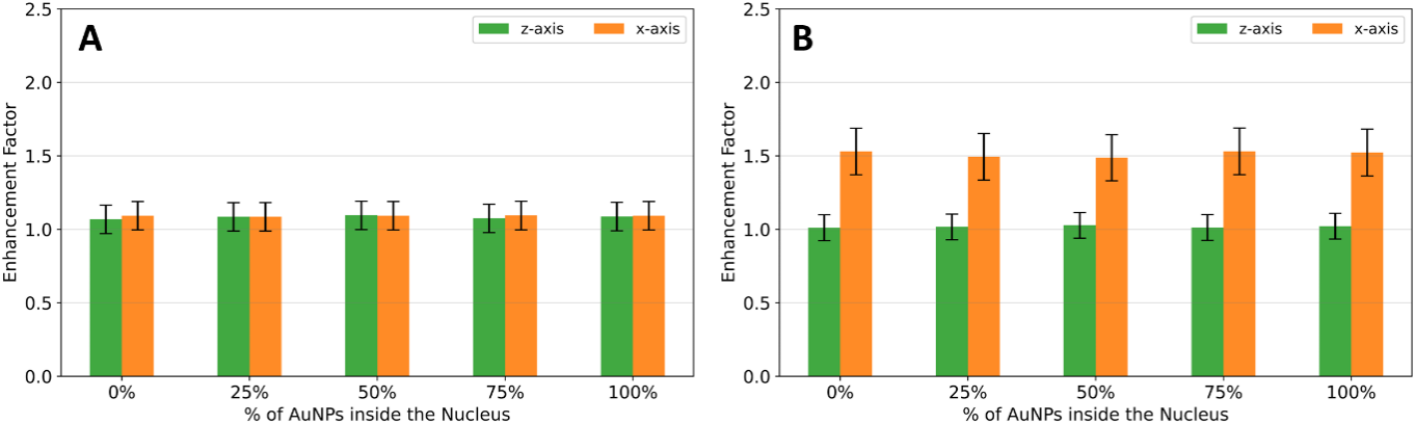
- Comparison of the survival fraction enhancement factor for a 0.5 Gy proton source irradiation along the z-axis (green) and x-axis (orange), for the **(A)** suspension and **(B)** adherent cell models

Regarding the adherent cell model, the SF at 0.5 Gy is highly superior in the case of the irradiation along the z-axis. The enhancement factor of survival at 0.5 Gy was also evaluated, being the results for both cell models illustrated in Fig. 9.

For the suspension cell model, the SF enhancement factor is identical for both irradiation setups, such as in the case of ^60^Co irradiation. This result is expected due to the spherical shape of the cells in suspension.

However, the difference in the SF enhancement factor is more pronounced in the case of adherent cells. The effect of irradiation and AuNPs is more significant in reducing the survival fraction when the cell model is irradiated along the x-axis, in accordance with the results obtained for ^60^Co irradiation.

These results indicate that the direction of irradiation plays a crucial role in determining the effectiveness of AuNPs in reducing survival fractions. Adherent cells are more sensitive to irradiation along the x-axis compared to the z-axis, probably due to differences in cellular orientation relative to the beam.

## Discussion

The main purpose of this comparative study was to verify the existence of radiosensitivity differences between adherent and suspended irradiated cells, using different irradiation setups, to optimize in vitro radiobiology experiments, particularly in the field of AuNPs as radiosensitization agents. Both cell models were exposed to ^60^Co and 14 MeV protons, with irradiation performed along different beam directions.

Recently, Daems et al. [23] highlighted the unique properties of AuNPs and their potential role in the future of medicine, both in diagnosis and therapy. The potential role of AuNPs in therapy is their ability to increase the dose deposition upon irradiation, via production of short-ranged secondary electrons around AuNPs able to, locally, release their energy, causing biological effects in cells. On one hand, the short-range electrons play a crucial role in biological enhancement. On the other hand, the results suggest an effective cell nucleus uptake. This is critical when nuclear DNA is considered the target for the biological outcome. Low-energy secondary electrons, with their shorter range, are more likely to induce DNA damage in the nucleus, leading to biological effects. Enea et al. [24] studied the physical-chemical properties of AuNPs in hepatic cell models, concluding that different size and shape, as well as the type of cellular model, greatly influence the AuNPs interaction with the biological environment. In the absence of AuNPs, the cell culturing type and the irradiation setup do not influence significantly the SF obtained for both irradiations. In the presence of AuNPs, one of the main conclusions of our work was the dependence of SF with the irradiation setup. The results indicated that suspension cells are more radioresistant than adherent cells to both types of radiation when irradiated along the x-axis, in the presence of AuNPs. The SF enhancement factor was also evaluated, demonstrating that, when the cells are adherent to the cell plate, AuNPs are more effective when irradiation is performed along the x-axis. Our work revealed that bio-effect modeling, regarding the use of AuNPs as radiosensitizers, is complex and highly dependent on cellular morphology, aligning with other studies found in the literature [25, 26].

## Conclusion

In this study, we examined the differences in radiosensitivity between adherent and suspended cells under various irradiation conditions. Our findings indicate that the direction of irradiation plays a crucial role in determining the effectiveness of AuNPs in reducing survival fractions. This study demonstrated that adherent and suspended cells exhibit differences in radiosensitivity under various irradiation conditions, emphasizing the impact of cell configuration on radiation response. Moreover, the results suggest that using cells in suspension minimizes the dependence of cell survival on beam direction, regardless of radiation quality.

These insights are essential for optimizing in vitro radiobiology experiments, particularly in the use of gold nanoparticles (AuNPs) as radiosensitizing agents and highlight the importance of considering both cellular morphology and beam direction in experimental design.

## Electronic supplementary material

Below is the link to the electronic supplementary material.

Supplementary Material 1

## Data Availability

All data generated or analyzed during this study are included in this published article and its supplementary information file.
