# Supplementary material for: Do cell culturing influence the radiosensitizing effect of gold nanoparticles: a Monte Carlo study
Source: EJNMMI Phys. 2025 Apr 18;12:41. doi: 10.1186/s40658-025-00746-3 (PMC12008099; doi:10.1186/s40658-025-00746-3)
Supplement: Supplementary file 1 — Supplementary Material 1 [file 40658_2025_746_MOESM1_ESM.docx]

**Supplementary Information**

***S1. Survival fraction in the absence of AuNPs***

PC3 human prostate cancer cells (ECACC, England, UK) were grown in RPMI supplemented with 10 % heat-inactivated fetal bovine serum (FBS) and 1% penicillin–streptomycin. Cells were grown at 37 °C in a humidified atmosphere of 5% CO_2_.

PC3 cells were seeded at an appropriate density in a 6 well-plate and allowed to attach overnight. Cells were then exposed to 0.5, 1, 2, 4, and 6 Gy. Cell irradiation was carried out by delivering γ-rays using a ^60^Co irradiator, PRECISA-22 (Graviner Manufacturing Company, Ltd., Buckinghamshire, UK). Briefly, Precisa-22 contains four sources placed inside stainless steel cylinders, where they move according to the action of a pneumatic system. The samples were placed on a support that rotates automatically to achieve a uniform dose rate of 1 Gy/min. The average dose rate in the wells of the cell plates was obtained the ionization chamber FC65P (IBA Dosimetry). The deviations from the wanted value of 1 Gy/min were very small, and the overall variation in the dose rate between plates was below 10 %.

Immediately, after irradiation, the culture medium was removed and cells returned to the incubator, in fresh culture medium, for 2 weeks. Colonies were fixed with methanol to glacial acetic acid (3:1) and stained with Giemsa (4%).

The Survival Fraction (SF) was obtained following the methodology described in [1]:

$$SF=\frac{number of colonies formed after treatment}{number of cells seeded}\times PE,$$

where $PE$ (Plating Efficiency) is defined as:

$$PE=\frac{number of colonies formed}{number of cells seeded}\times100\%$$

The survival fraction, in the absence of AuNPs inside PC3 cells, post-^60^Co irradiation, were fitted to the Linear Quadratic Model (Figure S1) to obtain the $\alpha$ and $\beta$ parameters (Table S1).


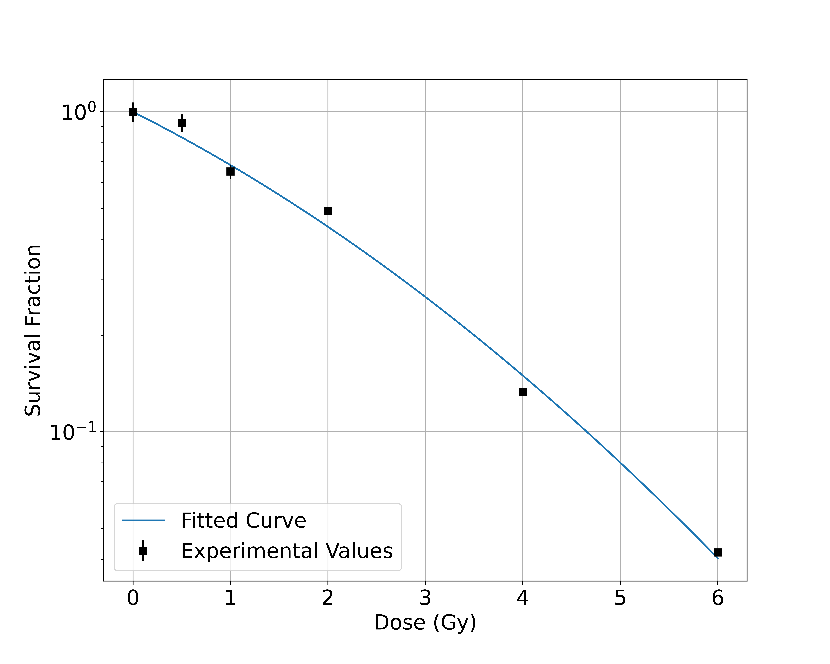


Figure S1: Fitted curve (blue line) and experimental data points of the survival fraction of PC3 cells (black squares) as a function of deposited dose. The results were calculated from independent biological replicates (n=3) and are given as the mean ± S.E.M.

Table S1: $\alpha$ and $\beta$ parameters.

| $\alpha(Gy^{-1}$) | $0.35\pm0.05$ |
| --- | --- |
| $\beta(Gy^{-2}$) | $0.03\pm0.01$ |

***S2. Orthogonal slices of the 3D cell models***

Orthogonal slices of the 3D cell models are illustrated in Figure S2. These slices represent key planes that help visualize the structure of the cell in three dimensions.

**
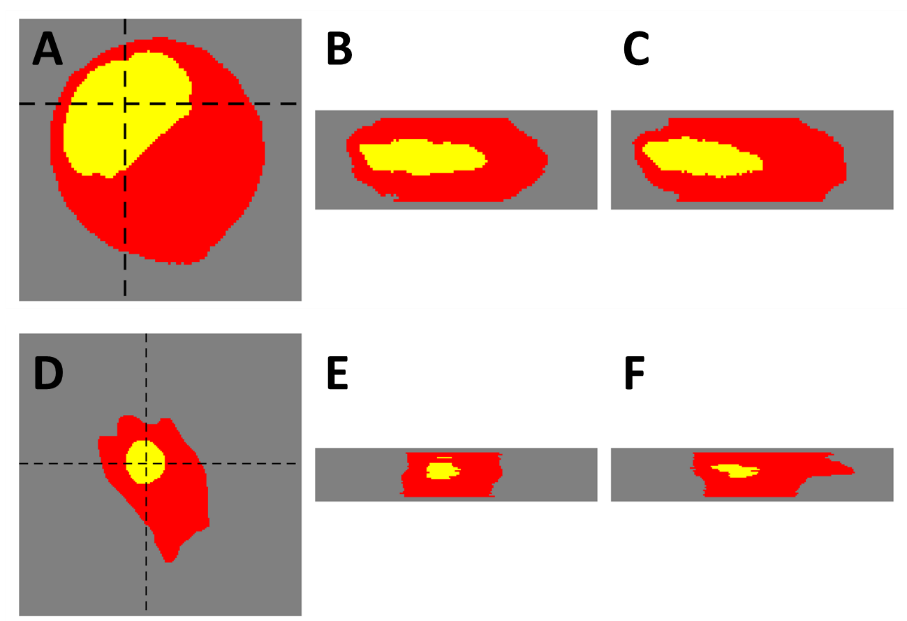
**

Figure S2. 3D cell geometry modeling. Top images show transverse (A) and longitudinal (B-C) cross-sections of the suspension cell model. Bottom images show transverse (D) and longitudinal (E-F) cross-sections of the adherent cell model. The cytoplasm (red) and nucleus (yellow) are segmented within a gray background. Dashed black lines in the transverse view indicate the slicing positions for the longitudinal views.

**References**

1. Franken NAP, Rodermond HM, Stap J, Haveman J, van Bree C. Clonogenic assay of cells in vitro. Nat Protoc. 2006;1:2315–9.
